# Evaluating an app for digital medical history taking in urgent care practices: study protocol of the cluster-randomized interventional trial ‘DASI’

**DOI:** 10.1186/s12875-023-02065-x

**Published:** 2023-04-27

**Authors:** Eva Maria Noack, Dagmar Zajontz, Tim Friede, Kai Antweiler, Eva Hummers, Tobias Schmidt, Lea Roddewig, Dominik Schröder, Frank Müller

**Affiliations:** 1 Department of General Practice, University Medical Center Göttingen, Humboldtallee 38, 37073 Göttingen, Germany; 2 Department of Medical Statistics, University Medical Center Göttingen, Humboldtallee 32, 37073 Göttingen, Germany; 3 Department of Performance, Neuroscience, Therapy and Health, MSH Medical School Hamburg, Kaiserkai 1, 20457 Hamburg, Germany

**Keywords:** Digitization, Application software, Out-of-hours practice, General practice, Diagnostic uncertainty, mHealth, Cluster randomized trial, Digital medical history taking, Urgent care

## Abstract

**Background:**

In out-of-hours urgent care practices in Germany, physicians of different specialties care for a large number of patients, most of all unknown to them, resulting in a high workload and challenging diagnostic decision-making. As there is no common patient file, physicians have no information about patients’ previous conditions or received treatments. In this setting, a digital tool for medical history taking could improve the quality of medical care. This study aims to implement and evaluate a software application (app) that takes a structured symptom-oriented medical history from patients in urgent care settings.

**Methods:**

We conduct a time-cluster-randomized trial in two out-of-hours urgent care practices in Germany for 12 consecutive months. Each week during the study defines a cluster. We will compare participants with (intervention group) and without app use (control group) prior to consultation and provision of the self-reported information for the physician. We expect the app to improve diagnostic accuracy (primary outcome), reduce physicians’ perceived diagnostic uncertainty, and increase patients’ satisfaction and the satisfaction with communication of both physician and patient (secondary outcomes).

**Discussion:**

While similar tools have only been subject to small-scale pilot studies surveying feasibility and usability, the present study uses a rigorous study design to measure outcomes that are directly associated with the quality of delivered care.

**Trial registration:**

The study was registered at the German Clinical Trials Register (No. DRKS00026659 registered Nov 03 2021. World Health Organization Trial Registration Data Set, https://trialsearch.who.int/Trial2.aspx? TrialID = DRKS00026659.

## Background

In Germany, ambulatory out-of-hours care is provided through walk-in practices run by the Associations of Statutory Health Insurance Physicians (in German: ‘Kassenärztliche Vereinigungen’, KV). Compared to general or family medicine practices, these urgent care clinics have limited diagnostic and therapeutic possibilities and are frequented by a heterogeneous group of patients of various ages and health complaints. Physicians almost exclusively provide care to patients they do not know from previous encounters. The physicians in these out-of-hour practices have various specialist backgrounds (i.e. not only specialized in general practice but, for example, in otolaryngology, gynecology, or dermatology) and professional experience. With time constraints and a high need for diagnostic and therapeutic clarification, the provision of care in out-of-hours consultations is considered highly complex and stressful [1]. Misdiagnosis, especially non-recognition of severe conditions that lead to (preventable) dangerous courses and subsequent delayed or inappropriate treatment, may occur as well as overuse of medical resources through unnecessary diagnostic and therapeutic measures or unnecessary referrals to emergency rooms or hospital admissions. Both are inefficient and increase costs for the health care system [2, 3].

Research findings suggest that only 70–85% of the diagnoses made by physicians of different specialties are accurate [4]. Errors related to delayed or missed diagnoses are most likely to harm patients or precipitate admission to hospital [5]. Some of these errors could be avoided if physicians had access to patients’ medical history so that they could include this information in their clinical assessment and decision-making [3, 6]. However, time for taking medical history is tight: A consultation in general practice in Germany takes 7.6 min on average [7]. During this time span, a physician obtains the patient’s medical history, responds to the patient’s concerns, performs a physical examination, considers different diagnoses, performs shared decision making, explains management, prescribe drugs and documents the visit in a note.

Collecting information on a patient’s medical history during the waiting time could help make better use of the limited consultation time and increase patients’ safety [8]. Studies have shown that questionnaires completed before the medical consultation enhance the safety of treatment and make it easier to assess individual risk constellation [9, 10]. The information provided by patients has been found to be more accurate and correct than those previously assessed by the physician [11]. In addition, a meta-analysis of randomized-controlled studies showed that patients were significantly more satisfied if they were asked about their expectations of the upcoming visit [12]. As this information is available to physicians and practice staff prior seeing the patient, it can be used for preparation, scheduling or even triage and may reduce documentation work. It can be assumed to be especially helpful in settings where a physician is providing care to a previously unknown patient.

Digital tools to obtain patients’ medical histories have been designed and evaluated for some specialties such as cardiology [13] or gastroenterology [14, 15] but only few are available for general practice [16, 17]. In a recently published scoping review on 18 digital tools designed for obtaining medical history from patients or caregivers [18], eight of the tools were not tailored to a specific patient subpopulation. While the research on these tools mainly comprise proof-of-concept and general usability, studies on patient experience and its impact on health outcomes are sparse.

### Study objectives

In the project “DASI” (acronym for ‘Digital assistierte Informationserfassung vor der Sprechstunde’; i.e. ‘Digitally supported system to obtain patient’s medical history before consultation’), we aim at piloting and evaluating a software application (app) developed for a structured symptom-oriented medical history taking among patients with acute medical complaints in general practice. While accuracy of obtained information and usability has been studied in another project [19], this study firstly addresses whether an app designed to obtain medical history before consultations in addition to regular medical history taking can improve diagnostic accuracy. Secondary, we aim to evaluate whether the use of the app prior to medical consultation increases patient satisfaction and reduces physicians’ perceived diagnostic uncertainty.

Within the framework of explorative data analysis (machine learning and data mining), complex prediction models are modeled based on the medical history data. The data is used to illustrate the potential use and opportunities of the software application for intelligent patient management, diagnostic and scientific purposes.

## Methods

### Software

We use an app that is designed to take the history of present illness from patients in general practice. The app covers the most common complaints in general practice. General practice touches on almost all medical specialties which entails that a respective tool must cover this broad and comprehensive field. A previous version of the app was originally developed for non-German-speaking patients by medical experts by aidminutes GmbH (Hamburg/Buchholz in der Nordheide, Germany) and piloted in a reception facility for refugees and asylum seekers [16, 20]. For this study, content and query structure were revised and further refined to meet requirements in out-of-hours practices. These adaptions were carried out jointly by aidminutes GmbH and experienced researchers from the Department of General Practice at the University Medical Center Göttingen. While the app is also available in other languages, the German language user interface is exclusively used for this study.

Patients choose their acute complaint(s) and are then guided through a query with questions related to their complaints, for example about specific symptoms, relevant pre-existing conditions, previous treatments, or medication. Depending on patients’ answers, follow-up questions regarding the current symptoms may be triggered. Questions and answers are phrased in plain language, the design is kept simple and navigation is intuitive, so that the app can be used by patients without prior instruction.

After completing the app, a structured summary of the medical history is compiled which can be transferred to the patient’s electronic medical record (EMR). The physicians can review this information before the patient encounter and can prepare themselves for the consultation and patients’ health needs.

For this study, participants used the app on iPad minis (6th generation, Apple, Inc, Cupertino, CA, USA).

### Study design

**Fig. 1:**
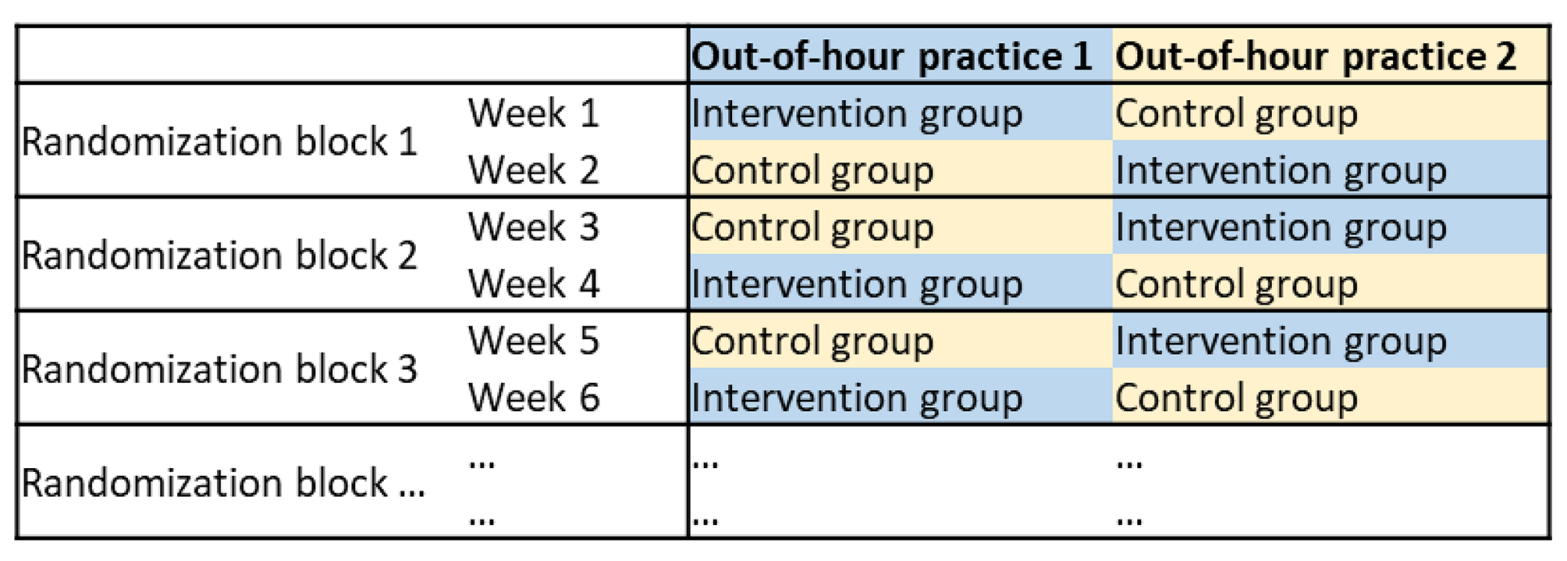
Example of two-week block randomization of participating out-of-hours practices for the first 6 weeks

To evaluate the effect of the developed app, a two-center, open-label, cluster-randomized trial (CRT) time-periods as clusters and groups under intervention or control condition will be conducted. In the intervention group, patients use the developed app in the waiting area before the encounter and the obtained information is available for the medical assistants and physicians before the consultation. In the control group, patients use the app after the consultation. The entered information is thus not available before the encounter. Clusters are defined as time periods of one week. In each week one practice is in the intervention group, the other one in the control group. The randomization of control and intervention group takes place before the start of the study in blocks of two weeks (see Fig. 1). Due to the two-weeks-block randomization, it is possible that a practice is in the control or intervention group for a maximum of two consecutive weeks.

The advantage of cluster randomization compared to patient-level randomization is that the data collection procedure remains the same during the service time. This prevents, for example, a physician from adapting their medical history taking of control group patients to those of intervention group patients, i.e. the cluster randomization minimizes the risk of contamination.

Data collection will take place for 12 consecutive months to control for seasonal effects. We will thus have aggregated data for two six-month periods for each intervention and control group from both centers.

#### Primary outcome

Diagnostic accuracy is the primary outcome of this study. For this purpose, the diagnoses of the attending physicians are compared with the diagnoses given by an expert committee consisting of three physicians experienced in general practice or family medicine. For each encounter, this expert committee will review the information obtained from the patient using the app (self-reported medical history), the documentation of the attending physician and the information provided by the patient about further treatment through a follow-up-survey, 14 to 21 days after the initial consultation. The committee is blinded whether a patient was assigned to intervention or control group. The decision of the committee is reached in a staged process: First, two physicians of the committee are asked to provide diagnose(s) for the encounter independently using the ICD-10-GM codes (10th revision of the International Statistical Classification of Diseases and Related Health Problems, German Modification) version 2019 by the World Health Organization (WHO; WHO Version 2019). If these diagnoses match, they will be compared to the diagnoses made by the physician of the out-of-hours practice. If the diagnoses of the expert committee physicians differ, a consensus on a diagnosis is reached with a third physician, which is then compared with the diagnosis made in the out-of-hours practice.

#### Secondary outcomes

Secondly, we hypothesize that the app reduces physicians’ perceived diagnostic uncertainty, increases patients’ satisfaction and the satisfaction with communication of both. Secondary outcomes of the clinical trial are therefore:

- the patients’ overall satisfaction,
- the patients’ satisfaction with the communication with the physician,
- the physicians’ satisfaction with the communication with the patient, and.
- the physicians’ perceived diagnostic uncertainty.

This data will be obtained using questionnaires for both participants and physicians. Participants’ overall satisfaction with the consultation will be assessed using questions from the standardized and validated questionnaire European Project on Patient Evaluation of General Practice Care Questionnaire (EUROPEP) in German language. This instrument comprises short questions on the quality of primary care which participants rate on a five-point Likert scale ranging from “poor” to “excellent” [21, 22]. The questions focusing on the communication for both, participants and physicians, are based on the Patient Satisfaction Questionnaire (PSQ) [23–25]. Each item is a secondary endpoint that will be analyzed individually.

#### Exploratory outcomes

The app compiles a short report (synopsis) of the patient’s answers for the physician. We will explore how well this summary matches the input. For this purpose, we will construct a parser that transforms the text of the synopsis into statistically accessible information. We will embed this information in a vector space and examine the correlation between the first principle components from this vector space and those from the recorded input data. We will also run cluster analyses on both data sources individually and compare the results. Further aspects for the exploratory outcomes are demographics (e.g. age, sex), medical complaints, as well as information about the usage of the app (e.g. number of questions asked, time needed to complete).

#### Big Data objectives

At the time of the final evaluation, there is an extensive standardized dataset of approximately 1,000 patients. A partial data set is to be evaluated in the context of a machine learning / data mining procedure. The aim is (a) to assess the usability of the data for further purposes (intelligent patient management and diagnostics) and to demonstrate this in a use case (e.g. predicting hospital admission), and (b) to characterize the usage process and to identify possibilities for improvement of the user interface.

### Setting

Patients are recruited in two out-of-hours urgent care walk-in practices in Lower Saxony, Germany. Both practices are run by a service company of the Association of Statutory Health Insurance Physicians of Lower Saxony (‘Kassenärztliche Vereinigung Niedersachsen’, KVN) and provide urgent care for patients with acute but not life-threatening complaints when other practices are closed. Both practices are located in hospitals that also have emergency rooms, but are not part of those hospitals.

Opening times of practice 1 are the following:

- Mondays, Tuesdays, and Thursdays from 7 p.m. to 10 p.m.,
- Wednesdays and Fridays from 3 p.m. to 11 p.m., and.
- Saturdays, Sundays, and public holidays from 8:30 a.m. to 11 p.m.

Opening times of practice 2 are the following:

- Mondays, Tuesdays, and Thursdays from 7 p.m. to 9 p.m.,
- Wednesdays and Fridays from 4 p.m. to 9 p.m., and.
- Saturdays, Sundays, and public holidays from 8 a.m. to 1 p.m. and 4 p.m. to 9 p.m.

Physicians work on a self-employed basis but are required to take shifts. They have different specialties and mostly provide care to patients they have not previously known. As there is no common patient file of different health care providers (e.g. medical specialists, physiotherapists) in Germany, physicians have no information about previous consultations or treatments of the patient.

Expenses are covered by the health insurances which German residents are legally required to have. The vast majority are covered through statutory health insurances, patients do not need to pay deductibles for medical consultations [26]. The minority of residents (11%) that are insured through a private insurance company receive a receipt and can later apply for reimbursement from the insurance company [26].

### Inclusion and exclusion criteria

Participating in the study is voluntary and certain inclusion and exclusion criterions are applied. Patients who meet the following criteria are eligible to participate in the study: (a) Seeking care in one of the participating out-of-hours practices due to acute discomfort, (b) aged 18 years or older, (c) able to declare written informed consent to participate in study. Patients who meet one of the following criteria cannot participate in the study: (a) younger than 18-year-old (legally minor), (b) patients in an apparent medical emergency, (c) patients who require immediate medical treatment, and (d) patients who are unable to consent. Recruited participants are informed by trained study nurses and need to declare consent to participate in the study.

### Data collection

Data collection takes place in the waiting area of the respective out-of-hours practices and is performed by study nurses.

**Fig. 2:**
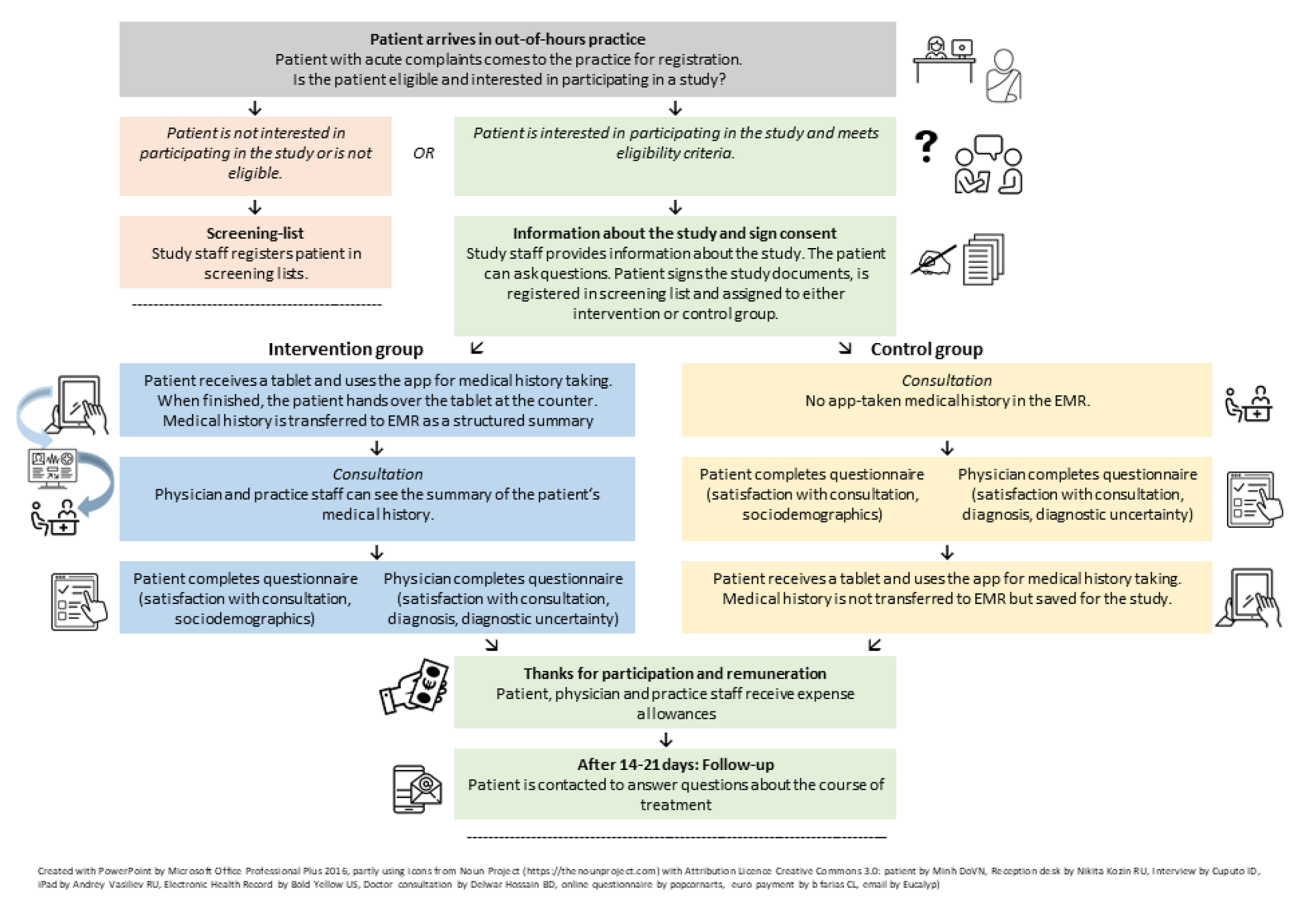
Inclusion of patients and data collection (EMR = electronic medical records)

The study nurse approaches patients, checks eligibility criteria, and provides interested patients with study information in the waiting area of the out-of-hours practices (Fig. 2). All approached patients are registered in screening lists. These lists include information about patients’ gender, birth year (requested from the patients) and, if applicable, the reason for non-participation (exclusion criterion, refusal or other). If a patient decides to take part in the study, written informed consent and the signed privacy policy statement are obtained. By declaring written informed consent, participants release the physician on duty from the medical confidentiality obligation. Therefore, the physicians must agree to the analysis of their documentations concerning the participating patients.

To contact participants for a follow-up survey after 14–21 days, contact information (phone number or email address) is requested. To be able to merge data sources, a pseudonym (identification number (ID)) is assigned to each enrolled participant.

After obtaining consent for participation, the patient is assigned to either intervention or control group according to the result of the cluster randomization.

#### Intervention group

In the intervention group, the participant then receives a tablet with the app installed. The participant completes the digital medical history in the waiting area. At the end of the survey, the app generates a report from the participants’ responses, which is subsequently transferred to the EMR via a scanned QR-code. This report is available to the physician before the consultation. No data remains stored on the device itself.

When they are next in line, the participant goes to the doctor’s office for consultation. After the consultation, both the participant and the physician are asked to fill out a short questionnaire. The participant will be asked questions about their satisfaction with the consultation (EUROPEP) and about communication (PSQ) and socio-demographic questions. These questions will be presented on the study tablets using the survey software Limesurvey. Subsequently, each participating patient receives a compensation of EUR 15.

The physician is asked to document the ICD-10-GM code (WHO Version 2019) of the (preliminary) diagnosis, to answer questions about their experience of uncertainty regarding this diagnosis and questions matching those that the participant is asked about communication. Additionally, we will collect data regarding the physicians’ specializations and experience (i.e. year of approbation and year of the first specialist examination).

After 14 to 21 days, we conduct a short follow-up to ask participants about their further treatment (e.g. by their family doctor, specialist treatment and hospital admissions). Participants may answer these questions via an online questionnaire (accessed using a personalized link which is sent per e-mail or text message) or by telephone (interview conducted by a study nurse).

#### Control group

After approving consent and data protection declaration, participants of the control group transition to their medical consultation and will use the app for medical history after the encounter. Information gathered from the app is not transferred to the EMR. Therefore, contrary to interventional group, for control participants, physicians do not have additional information collected by the app. After the consultation, the participant and the physician are asked to complete questionnaires as described for the intervention group. Remuneration and follow-up procedure are then identical to the intervention group.

### Data management

Once transferred to the EMR, the information on the participants’ medical history collected by the app is available to the practice staff and this data is linked to the patient file. Pseudomized app data and questionnaire data are available to the project partners. The paper-based declarations of consent and the confirmation of receipt of the expense allowance containing personal information cannot be linked to this data. The contact details (telephone number and/or e-mail) are recorded together with the ID but stored separately from the consent forms and destroyed after expiration of the agreed time period of 21 days. Then, the data can no longer be traced back to study participants. Collected data is treated confidentially. The research team is aware of data privacy regulations and committed to data protection. Data and information are not shared with other authorities or third parties. Data evaluation and publication of results is exclusively anonymous. All study data will be destroyed or deleted 10 years after the end of the study.

### Sample size estimation

The sample size is driven by the primary outcome diagnostic accuracy. For the planning of the study, we assume the diagnostic accuracy will increase by 10% points, with an expected current diagnostic accuracy of 70–80% [4, 27]. In a design with randomization of individual participants, a case number of 335 participants per group (670 participants in total) gives a power of 90% at a two-sided significance level of 5% assuming diagnostic accuracies of 75% and 85% in the control and intervention groups, respectively.

To correct for the randomization of clusters (time periods), the number of cases is multiplied by the design effect. The design effect is a function of the size of the clusters and the correlation within the clusters (ICC). We originally assume a cluster size of 36 participants per cluster of two weeks in both practices overall and an ICC of 0.01. The difference between the two practices is accounted in the analysis model by a fixed-effect co-factor. This results in a design effect of 1.35. In addition, we count for about 5% drop-out of the participants. This results in a rounded total number of cases of n = 1,000. In the execution of the study, we reduced the duration of each cluster to one week and thereby increased the number of clusters from 26 to 52. This reduced the number of cases needed (originally 952) slightly to 825. This adjustment does not reduce the power of the study and is determined to be convenient in its implementation. The duration of the study is kept at 12 months to cover all seasons.

### Statistical design

We will use descriptive statistics to analyze patient characteristics (i.e. demographics, medical complaints) of intervention and control group. Furthermore, the groups will be compared according to characteristics of the recruitment centers.

The primary outcome “diagnostic accuracy” defines the agreement of a given diagnosis with the determined correct diagnosis. The primary evaluations of the binary endpoint of an adequate diagnosis are performed by a generalized linear mixed-effects model (hierarchical or multilevel model) with logit link function. The group (intervention versus control) and the respective out-of-hours practice are included as fixed effects, and the time period (cluster) as a random effect. The random effect is assumed to be normally distributed on the logit scale. The expected value is 0. The variance is unknown and estimated from the data. The odds ratio for the intervention effect will be reported together with its 95% confidence interval and a p-value for the null hypothesis that the intervention does not increase the diagnostic accuracy. Subgroup analyses will explore whether practice characteristics such as experience or specialization of the physicians have an influence on the strength of the intervention effect. This will be done by extending the model described above. Specifically, the respective practice characteristic as well as its interaction with the intervention effect will be included in the hierarchical model. With regard to the secondary endpoints, the individual items of the respective questionnaires are first presented within the framework of descriptive statistics (median, max, min, percentiles). Differences between the intervention and control group are analyzed for each item of the questionnaires using non-parametric methods. Except for the primary endpoints all endpoints are considered exploratory. Therefore, p-values will not be adjusted for multiplicity. Further explorative evaluations will be conducted using machine learning and data mining procedures: methods that are known to work well with a medium sample size will be used for classifications. These methods should have the potential to capture the structure of the underlying associations. We assume that some effects of inquired symptoms on the suspicion of disease severity are additive and individually assessable while others are more likely to conform to a relation like “at least k of n criteria have to be met and none of the following contra-indications” and are more specific to single diseases. We use elastic nets for classifications, because they perform well with the first class of symptom effects even when the number of inquired symptoms is larger than the number of patients. We will also use random forests, because they structurally have some similarity to decision tree like associations that can handle the second class of symptoms. We will utilize the results from these methods to artificially expand the data set for (or to pre-train) more flexible algorithms, such as support vector machines and artificial neural networks. These will then be used to search for more complicated associations. Medical history information, demographic information, treatment information (e.g. diagnosis, examination findings), and event-sourcing information (tracking of the app-usage of individual patients, e.g. time needed to complete) will be used as input variables. In addition, cluster, association and regression analyses will be performed. Results will be visualized and interpreted. Test data from the overall evaluation will be used to determine sensitivity, specificity, and accuracy of prediction models.

## Discussion

There have been several attempts to develop and implement digital tools as well as paper-based questionnaires to obtain information on health concerns from patients before their encounter. While many aim to improve quality of medical care, ease documentation and enhance work efficiency, these effects have hardly been studied [18]. The purpose of this study is to generate evidence on the effect of an app for medical history taking in urgent care practices in Germany using a rigorous study design. In these urgent care practices, physicians with various specialist backgrounds have to assess complaints they normally do not see in their daily practice. The app provides them with a structured medical history that might prevent them from making diagnostic errors which might lead to insufficient and ineffective care.

Along the project, we will also gain experience on the implementation process and potential difficulties, e.g. with the technical infrastructure or users. This is important seeing that in Germany the level of digitization is low in the healthcare sector, both compared internationally and with other sectors within Germany. While physicians in Germany are increasingly positive about opportunities to improve medical care through the greater use of digital technologies [28], the difficulty of proving benefits and the lack of interoperability are considered to be major obstacles [29]. Regarding the latter aspect, it is a major challenge to make the information transferred into patients’ EMR to be of use in the long term. Up to now, it remains on the information system of the physicians on duty but cannot be exchanged with other systems or transferred to the not-yet-used individual electronic patient file. As a step towards rendering the data interchangeable we aim at developing and publishing an open digital format for symptom-oriented health inventory data.

As for limitations, we face several challenges that might introduce bias: Data collection takes place during COVID-19 pandemic with altered contact patterns in medical care. Patients with signs of a respiratory infection, for example, are sometimes not allowed to enter the waiting areas and might be underrepresented in this study. Even though elderly people are increasingly participating in the digital world [30], their usage behavior differs from that of younger people [31]. Elderly people might be less inclined to take part in a study with a digital intervention; their complaints, diagnoses and treatment courses might be underrepresented in the study. Patients with a private health insurance are less likely to seek medical care in the out-of-hour practices. Furthermore, patients with limited German-language proficiency cannot take part in this study as we focus on German speaking patients. Of note, many patients with limited German language proficiency are treated in these practices. As professional interpreter are not regularly covered through statutory health insurance and only a minority of physicians in Germany offer consultations in languages other than German or English [32], further studies should be considered to investigate the benefits for migrant and refugee patients with limited German language skills.

## Data Availability

Not applicable.

## List of abbreviations

App: software application
CRT: cluster-randomized trial
EMR: Electronic medical records
EUROPEP: European Project on Patient Evaluation of General Practice Care Questionnaire
DASI: Digital assistierte Informationserfassung vor der Sprechstunde (Digitally supported system to obtain patient’s medical history before consultation)
G-BA: Gemeinsamer Bundesausschuss (Federal Joint Committee)
ICC: Intra class correlation
ICD-10-GM: 10th revision of the International Statistical Classification of Diseases and Related Health Problems, German modification
ICH-GCP: International Conference on Harmonization Good Clinical Practice
ID: Identification number
KV: Kassenärztliche Vereinigung (Association of Statutory Health Insurance Physicians)
KVN: Kassenärztliche Vereinigung Niedersachsen (Association of Statutory Health Insurance Physicians of Lower Saxony)
PSQ: Patient Satisfaction Questionnaire
WHO: World Health Organization

## References

[1] Leutgeb R, Frankenhauser-Mannuß J, Scheuer M, Szecsenyi J, Goetz K (2018). Job satisfaction and stressors for working in out-of-hours care – a pilot study with general practitioners in a rural area of Germany. BMC Fam Pract.

[2] Kostopoulou O, Delaney BC, Munro CW (2008). Diagnostic difficulty and error in primary care–a systematic review. Fam Pract.

[3] Bhise V, Rajan SS, Sittig DF, Morgan RO, Chaudhary P, Singh H (2018). Defining and measuring diagnostic uncertainty in Medicine: a systematic review. J Gen Intern Med.

[4] Semigran HL, Levine DM, Nundy S, Mehrotra A (2016). Comparison of Physician and Computer Diagnostic Accuracy. JAMA Intern Med.

[5] Sandars J, Esmail A (2003). The frequency and nature of medical error in primary care: understanding the diversity across studies. Fam Pract.

[6] Alam R, Cheraghi-Sohi S, Panagioti M, Esmail A, Campbell S, Panagopoulou E (2017). Managing diagnostic uncertainty in primary care: a systematic critical review. BMC Fam Pract.

[7] Irving G, Neves AL, Dambha-Miller H, Oishi A, Tagashira H, Verho A, Holden J (2017). International variations in primary care physician consultation time: a systematic review of 67 countries. BMJ Open.

[8] Sherwin HN, McKeown M, Evans MF, Bhattacharyya OK (2013). The waiting room “wait. Can Fam Physician.

[9] Emery JD, Reid G, Prevost AT, Ravine D, Walter FM (2014). Development and validation of a family history screening questionnaire in australian primary care. Ann Fam Med.

[10] Carroll JC, Campbell-Scherer D, Permaul JA, Myers J, Manca DP, Meaney C (2017). Assessing family history of chronic disease in primary care. Can Fam Physician.

[11] Porter SC, Forbes P, Manzi S, Kalish LA (2010). Patients providing the answers: narrowing the gap in data quality for emergency care. Qual Saf Health Care.

[12] Kinnersley P, Edwards A, Hood K, Cadbury N, Ryan R, Prout H (2007). Interventions before consultations for helping patients address their information needs. Cochrane Database Syst Rev.

[13] Brandberg H, Kahan T, Spaak J, Sundberg K, Koch S, Adeli A (2020). A prospective cohort study of self-reported computerised medical history taking for acute chest pain: protocol of the CLEOS-Chest Pain Danderyd Study (CLEOS-CPDS). BMJ Open.

[14] Almario CV, Chey W, Kaung A, Whitman C, Fuller G, Reid M (2015). Computer-generated vs. physician-documented history of present illness (HPI): results of a blinded comparison. Am J Gastroenterol.

[15] Almario CV, Chey WD, Iriana S, Dailey F, Robbins K, Patel AV (2015). Computer versus physician identification of gastrointestinal alarm features. Int J Med Inform.

[16] Kleinert E, Müller F, Kruse S, Furaijat G, Simmenroth A (2021). Nutzbarkeit digitaler Anamnesehilfen für nicht-deutschsprachige Patienten in der allgemeinärztlichen Sprechstunde. [Usability and efficiency of a Digital Communication Assistance Tool to Obtain Medical History from Non-German-Speaking Patients]. Gesundheitswesen.

[17] Zakim D (2016). Development and significance of automated history-taking software for clinical medicine, clinical research and basic medical science. J Intern Med.

[18] Berdahl CT, Henreid AJ, Pevnick JM, Zheng K, Nuckols TK (2022). Digital Tools designed to obtain the history of Present Illness from Patients: scoping review. J Med Internet Res.

[19] Albrink K, Joos C, Schröder D, Müller F, Hummers E, Noack EM (2022). Obtaining patients’ medical history using a digital device prior to consultation in primary care: study protocol for a usability and validity study. BMC Med Inform Decis Mak.

[20] Furaijat G, Kleinert E, Simmenroth A, Müller F (2019). Implementing a digital communication assistance tool to collect the medical history of refugee patients: DICTUM Friedland - an action-oriented mixed methods study protocol. BMC Health Serv Res.

[21] Wensing M, Vedsted P, Kersnik J, Peersman W, Klingenberg A, Hearnshaw H (2002). Patient satisfaction with availability of general practice: an international comparison. Int J Qual Health Care.

[22] Klingenberg A, Bahrs O, Szecsenyi J. Wie beurteilen Patienten Hausärzte und ihre Praxen? Deutsche Ergebnisse der europäischen Studie zur Bewertung hausärztlicher Versorgung durch Patienten (EUROPEP) [How do patients evaluate general practice? German results from the European Project on Patient Evaluation of General Practice Care (EUROPEP)]. [How do patients evaluate general practice? German results from the European Project on Patient Evaluation of General Practice Care (EUROPEP)]. Zeitschrift fur ärztliche Fortbildung und Qualitätssicherung. 1999;93:437–45. 10519193

[23] Blanchard CG, Ruckdeschel JC, Fletcher BA, Blanchard EB (1986). The impact of oncologists’ behaviors on patient satisfaction with morning rounds. Cancer.

[24] Ong L, Visser M, Lammes FB, Haes J. de. Doctor–Patient communication and cancer patients’ quality of life and satisfaction. Patient Education and Counseling. 2000;41:145–56. doi:10.1016/S0738-3991(99)00108-1. 10.1016/s0738-3991(99)00108-1 12024540

[25] Zandbelt LC, Smets EMA, Oort FJ, Godfried MH, de Haes HCJM (2004). Satisfaction with the outpatient encounter: a comparison of patients’ and physicians’ views. J Gen Intern Med.

[26] Busse R, Blümel M, Knieps F, Bärnighausen T (2017). Statutory health insurance in Germany: a health system shaped by 135 years of solidarity, self-governance, and competition. Lancet.

[27] Graber ML (2013). The incidence of diagnostic error in medicine. BMJ Qual Saf.

[28] Haserück A, Digitalisierung. Ärzteschaft sieht mehrheitlich vor allem Chancen. Deutsches Ärzteblatt;2022:A1778.

[29] Nohl-Deryk P, Brinkmann JK, Gerlach FM, Schreyögg J, Achelrod D (2018). Hürden bei der Digitalisierung der Medizin in Deutschland – eine Expertenbefragung. [Barriers to Digitalisation of Healthcare in Germany: a survey of experts]. Gesundheitswesen.

[30] Deutsches Institut für Vertrauen und Sicherheit im Internet (DIVSI). DIVSI Ü60-Studie. Die digitalen Lebenswelten der über 60-Jährigen in Deutschland. Hamburg; 2016.

[31] Initiative D. e. V. D21-Digital-Index 2018 / 2019. Berlin; 2019.

[32] Müller F, Holman H, Hummers E, Schröder D, Noack EM (2022). Multilingual competencies among ambulatory care providers in three german Federal States. BMC Prim Care.

